# Test–Retest Reliability and Concurrent Validity of the 3 m Backward Walk Test under Single and Dual-Task Conditions in Women with Fibromyalgia

**DOI:** 10.3390/jcm12010212

**Published:** 2022-12-27

**Authors:** Juan Luis Leon-Llamas, Santos Villafaina, Alvaro Murillo-Garcia, Francisco Javier Domínguez-Muñoz, Narcis Gusi

**Affiliations:** 1 Grupo de Investigación Actividad Física y Calidad de Vida (AFYCAV), Facultad de Ciencias del Deporte, Universidad de Extremadura, 10003 Caceres, Spain; 2 Departamento de Desporto e Saúde, Escola de Saúde e Desenvolvimento Humano, Universidade de Évora, 7004-516 Évora, Portugal; 3 International Institute for Innovation in Aging, University of Extremadura, 10003 Caceres, Spain

**Keywords:** reproducibility, assessment, chronic pain, activities of daily living, mobility

## Abstract

Background: Previous studies have reported good test–retest reliability for the 3 m backward test (3MBWT) in different populations. However, reliability of the 3MBWT has not been studied in fibromyalgia (FM) under single and dual-task conditions; Methods: A total of 21 women with FM participated in this study. Participants completed the Revised Fibromyalgia Impact Questionnaire and two physical fitness tests: the 3MBWT and the Timed Up and Go (TUG). The dual-task condition consisted of subtracting two by two while performing the test, starting from a random number less than 100; Results: Values showed that the 3MBWT can be considered reliable under single and dual-task conditions when measured with both a manual stopwatch and a Chronopic automatic stopwatch. A strong concurrent validity was shown of 3MBWT and TUG results in the test and retest and the different devices. The relationship between the performance of the 3MBWT in test and retest conditions under single and dual-task conditions measured with different devices and the impact of the disease were high; Conclusions: The 3MBWT is a reliable tool under the single and dual-task conditions in women with FM. It shows higher reliability values when time is taken using a Chronopic. This test also shows high concurrent validity with the TUG test. Its performance is related to the impact of the disease.

## 1. Introduction

Fibromyalgia (FM) is a chronic disease that is characterized by chronic widespread, diffuse, and persistent musculoskeletal pain, often accompanied by other symptoms, such as fatigue, sleep disorders, mood disturbance, anxiety, depression, cognitive problems, low physical activity, and balance problems [1,2,3]. All these symptoms have an important influence on the activities of daily living [4] and tend to reduce the health-related quality of life in this population [5]. It is estimated that FM affects 0.2% to 6.6% of the general population and mainly women over 50 years old [6].

One of the ten most debilitating symptoms of FM is balance impairments, which is experienced by 45% of this population [7]. Moreover, people with FM usually report nonspecific postural balance disorder, an increased prevalence of falls [8], a reduced performance in mobility [9,10,11], a higher risk of falling [12,13,14], and, therefore, a lower performance on balance tests [12,14]. In addition, gait disturbances [15] that are influenced by attention and executive function [16] have been also detected.

One of the most important objectives that a rehabilitation or training program should follow is to increase the individual’s performance to minimize the risks associated with the condition. Therefore, previous studies have evaluated the physical fitness of people with FM using different tests to assess flexibility [17], endurance [10], strength [10,17,18,19], balance [9], or mobility [11,17]. Among the physical fitness tests used to assess functional mobility, the Timed Up and Go test (TUG) has been used in different populations [20,21], including FM [9,11,17]. This test involves walking forward, balance, and turning tasks. Nevertheless, walking backward, which is not contemplated in the TUG test, is more complex and requires higher neuromuscular and proprioceptive control [22]. Moreover, it is a task that can occur in everyday life situations, such as opening a door, avoiding an obstacle, or backing up to a chair [23]. Additionally, walking backward is considered a more sensitive measure for assessing mobility and balance deficits [24,25]. In this regard, Carter et al. [23] proposed the 3 m walking backward test (3MBWT). This is a clinical tool developed in healthy older adults to identify the risk of falling that appears to be more accurate or equal to other existing tests such as TUG, Five Times Sit-to-Stand, and Four Square Step Test. Regarding the 3MBWT, it has shown high test–retest reliability and validity in the stroke population [26], community-dwelling older adults [27], multiple sclerosis [28,29], and patients with advanced knee osteoarthritis [30]. However, this test has yet to be studied in people with FM. Interestingly, it could become an important clinical tool due to the characteristics of this population since it is essential to perform a functional assessment of mobility and balance to aid in diagnosing and managing the disease.

Due to the similarities to real-life conditions and activities of daily living requirements [31], previous studies have included a simultaneous cognitive task (dual-task paradigm). Therefore, assessing these activities is essential in clinical and ecological settings since they require significant attention and executive processes [31]. In this regard, people with FM have exhibited a considerable impairment in dual-task performance compared to healthy controls [32,33,34]. Furthermore, the reliability of the chair stand test [18], 10-m walking test [11], TUG [11], and arm curl test [18] under dual-task conditions have been explored for people with FM. Nevertheless, the reliability of walking backward while performing a cognitive task has yet to be assessed. This issue is crucial since healthcare professionals and researchers can better understand an individual’s symptoms and develop a more effective treatment plan to address their specific needs.

To our knowledge, previous investigations have not explored the reliability and validity of the 3MBWT in people with FM. Therefore, this study aimed to analyze the test–retest reliability of the 3MBWT under single and dual-task conditions. As a secondary objective, we also aimed to evaluate the test–rest reliability using different instruments (stopwatch and Chronopic). Lastly, we also aimed to assess the concurrent validity of the TUG and 3MBWT as well as the relationship between the 3MBWT test and the impact of the disease. We hypothesized that good test–retest reliability values would be obtained with both Chronopic and stopwatch, with higher scores when using a Chronopic, as previous studies suggested [9,11]. Additionally, a high concurrent validity between the 3MBWT and TUG test would be obtained considering the results reported in previous research [26,27,28,29], and a significant correlation between the 3MBWT and the impact of the disease would be observed.

## 2. Materials and Methods

### 2.1. Participants

Twenty-one women with FM were enrolled in this cross-sectional study. The sample size and statistical power were calculated using the PASS software (version 11.0; PASS; Kaysville, Utah). In this regard, with two samples per participant, there is a 98% power to detect an intra-class correlation of 0.95 under the alternative hypothesis when the intraclass correlation under the null hypothesis is 0.75, using an F-test with a significance level of 0.05.

The participants fulfilled the following inclusion criteria for this study: (a) to be a female between 35 and 65 years old, (b) to be diagnosed with FM by a rheumatologist according to the criteria established by the American College of Rheumatology [35], and (c) to understand the physical fitness protocols. Participants were excluded if they: (a) were pregnant, (b) were enrolled in another clinical trial or research that could impact the results, and (c) had any condition where exercise is contraindicated.

All the participants gave written informed consent. The Research Ethics Committee of the University of Extremadura approved the protocols of the current study (approval reference: 51/2021).

### 2.2. Procedure

The Spanish version of the Revised Fibromyalgia Impact Questionnaire (FIQR) was administered [36]. This instrument is composed of 21 items divided into three domains (function, overall impact, and symptoms). The maximum score is 100, which corresponds to the worst overall impact. In addition, age and anthropometric measurements were acquired using a Tanita Body Composition Analyzer BC-418 MA (Tanita Corp., Tokyo, Japan).

The 3MBWT and (2) the TUG were performed under single and dual-task conditions. The dual-task condition consisted of subtracting two by two (a random number lower than 100) while performing the physical fitness tests.

The 3 m Backward Walk Test (3MBWT) was performed according to the procedure proposed by Carter et al. [23]. A distance of three meters was measured with black tape establishing the start and finish. Participants were asked to place their heels on the start mark. Then, they had to walk backward as fast and safely as possible at the “go” signal. Running was not allowed, and they could look behind themselves if they wished.

In the Timed Up and Go (TUG) test, participants had to get up from a chair without armrests, walk a distance of 3 m without running, turn around a cone, walk back to the chair, and sit down [37].

Simultaneous stopwatch and automatic timer records were obtained. For the TUG, the Chronopic (Chronojump, BoscoSystem^®^, Barcelona, Spain) time was obtained using a DIN A4-sized contact platform placed on the back of the chair, which was used to open and close the circuit to obtain the test time [9,11]. For the 3MBWT a DIN A2-sized contact platform on the start line combined with a photocell on the end line was used. Physical tests were repeated after seven days to avoid learning effect [11,18,38,39]. Participants performed three trials for each condition (single and dual-task), and the order of TUG test and 3MBWT was randomized.

### 2.3. Statistical Analysis

Statistical analysis was conducted using the Statistical Package for the Social Sciences (SPSS, version 24.0; IBM Corp., Armonk, NY, USA) software. Based on data provided by the Shapiro–Wilk test, parametric tests were employed. The statistical significance was established at the *p* ≤ 0.05 level. To estimate the intraclass correlation coefficient (ICC) and its 95% confidence intervals of the 3MBWT in the single and dual-task conditions at test and retest times, the 3,1 (Two-way mixed effects, consistency, single rater/measurement) model was used following the recommendations by Weir [40] and Koo [41]. Regarding the ICC classification, an ICC value lower than 0.50 indicates “poor” reliability, an ICC value between 0.50 and 0.75 indicates “moderate” reliability, an ICC value between 0.75 and 0.90 indicates “good” reliability, and an ICC value higher than 0.90 indicates “excellent” reliability. This ICC classification was interpreted according to the guideline proposed by Koo [41].

The standard error of measurement (SEM) was calculated using the following formula:

(1) $$\mathrm{SEM}=\mathrm{SD}\;\times \sqrt{1-\mathrm{ICC}}$$

The minimal detectable change (MDC) was obtained according to the formula:

(2) $$\mathrm{MDC}=1.96\times \mathrm{SEM}\;\times \sqrt{2}$$

The SEM and MDC were expressed as a percentage according to the following formula, SEM% or MCD%=(SEM or MCD/mean)×100, where the mean is the average of the test and retest.

To identify the level of agreement between the test and retest, and the measuring devices in the 3MBWT under single and dual-task conditions, Bland–Altman plots were performed [42].

The Pearson’s product–moment correlation coefficient (r) was used to explore the concurrent validity comparing the 3MBWT and the TUG. Finally, the relationship between 3MBWT and the impact of the disease was also analyzed through the total value of the FIQR. Cohen’s recommendations [43] were followed to interpret the correlation coefficient. A score ≥ 0.5 was strong, moderate if the score was between 0.5 and 0.35, and poor if the score was ≤0.35.

## 3. Results

A total of 21 women with FM from a local association participated in this study. The main characteristics of the participants are shown in Table 1.

Table 2 shows the relative reliability (ICC) and absolute reliability (SEM and MDC) of the performance obtained in the 3MBWT, under the single and dual-task conditions in both test and retest with the different devices. Following the recommendations by Koo et al. [41], the 95% confidence intervals of the ICC were used to interpret the reliability values. Regarding the single condition, “poor” to “good” and “moderate” to “excellent” reliability values were obtained for the stopwatch and Chronopic, respectively. On the other hand, in the dual-task condition, the reliability values for the stopwatch were “moderate” to “excellent” and for the Chronopic were “good” to “excellent”.

Reliability values obtained by comparing the different devices in both the single and dual-task conditions in the test and retest are shown in Table 3. Taking into account the 95% confidence intervals of the ICC, a reliability value of “good” to “excellent” was obtained for the test, and an “excellent” value was obtained for the retest in the single and dual-task condition.

**Figure 1:**
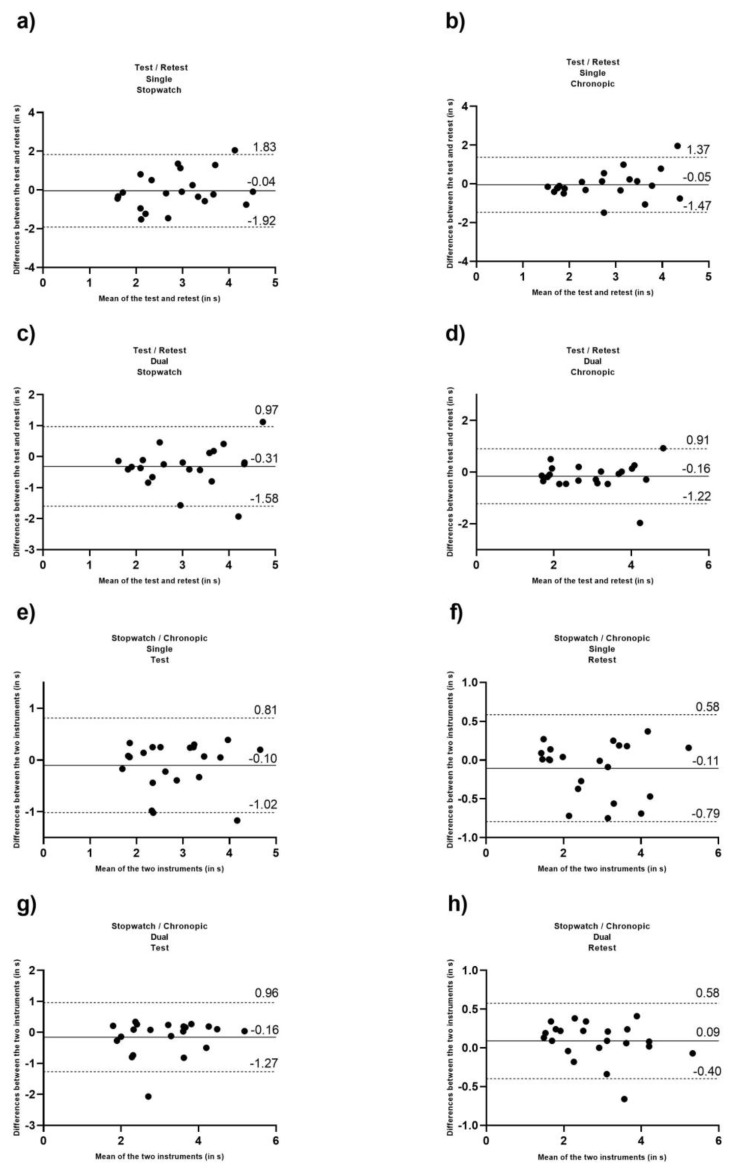
(**a**) differences between test and retest vs. the mean of the two measurements under the single condition using a stopwatch; (**b**) differences between test and retest vs. the mean of the two measurements under the single condition using a Chronopic; (**c**) differences between test and retest vs. the mean of the two measurements under the dual-task condition using a stopwatch; and (**d**) differences between test and retest vs. the mean of the two measurements under the dual-task condition using a Chronopic; (**e**) differences between stopwatch and Chronopic vs. the mean of the two measurements under the single condition in test; (**f**) differences between stopwatch and Chronopic vs. the mean of the two measurements under the single condition in retest; (**g**) differences between stopwatch and Chronopic vs. the mean of the two measurements under the dual-task condition in test; and (**h**) differences between stopwatch and Chronopic vs. the mean of the two measurements under the dual-task condition in retest.

Figure 1 shows the Bland–Altman plots of the times obtained by stopwatch and Chronopic in test and retest in the single and dual-task conditions, and the times obtained between the two devices in both the single and dual-task conditions in the test and retest, respectively.

Table 4 shows the concurrent validity analysis results of 3MBWT and TUG test. All correlation values obtained were classified as strong [43] in the test and retest and the different devices.

Finally, the relationship between the performance of the 3MBWT in test and retest conditions under single and dual-task conditions measured with different devices and the impact of the disease were strong, excepting the 3MBWT in test condition under single condition measured with a stopwatch (r: 0.488), which was moderate. In analyzing the dimensions that make up the FIQR questionnaire, strong correlations were obtained between the “symptoms” dimension and all the conditions and devices. Similarly, strong correlations were also obtained between the dimension “overall impact” except for the single and dual-task conditions in the test period, measured with the stopwatch and the Chronopic, respectively, where moderate correlations were reported. As for the dimension “function,” only moderate correlations were found in the single condition in the retest and test periods, measured with a stopwatch and Chronopic, respectively. Results are shown in Table 5.

Figure 1 Bland–Altman plots of the times obtained by stopwatch and Chronopic in test and retest under the single and dual-task conditions and Bland–Altman plots of the times obtained between the two devices under the single and dual-task conditions in the test and retest.

## 4. Discussion

This study aimed to investigate the test–retest reliability and concurrent validity of the 3MBWT in women with FM under single and dual-task conditions. We also aimed to investigate the agreement between a manual stopwatch and a Chronopic automatic stopwatch. In order to provide clinical and objective directions, the SEM, MDC, and Bland–Altman plots were reported. Generally, good reliability values were obtained for the 3MBWT test when measured with a stopwatch and Chronopic in both single and dual-task conditions. Similarly, the 3MBWT and the TUG achieved good concurrent validity. Lastly, performance between 3MBWT and the impact of the disease were analyzed, and consistent relationships were found between both.

In the present study, walking backward has been analyzed because it is a complex task that requires high neuromuscular and proprioceptive control [22]. Walking backward usually occurs in activities of daily living, such as opening a door, avoiding an obstacle, or backing up to a chair [23]. Therefore, it is a sensitive measure for assessing mobility and balance deficits [24,25]. In people with FM, performing a functional assessment of mobility and balance is essential to manage the disease. However, one of the main tests used to evaluate these characteristics in this population is the TUG [9,11,17]. However, this test does not include backward gait. In this sense, we consider it relevant to analyze the psychometric properties of the 3MBWT in people with FM since previous studies have focused on other populations obtaining good reliability and validity values [26,27,28,29]. Therefore, the 3MBWT is a tool that can be used elsewhere in the clinical and research context. Moreover, due to the fact that more than one task is performed at the same time in activities of daily living, and the impairment in dual-task ability detected on people with FM [32,44,45], we decided to incorporate the dual-task paradigm in this cross-sectional study.

Results showed that the 3MBWT could be considered reliable under single and dual-task conditions when measured with both a stopwatch and a Chronopic. However, it is necessary to consider the ICC fluctuation range [41]. Nevertheless, the data presented in the Bland–Altman plots (Figure 1) showed that most of the 3MBWT values were close to the mean of the test–retest differences in both single and dual-task conditions, having a bias close to zero and a reduced variability seeing the limits of agreement. Therefore, there is a high level of agreement between the test–retest measures evaluated in the 3MBWT since the values obtained provide consistent results. The reported ICC in the single condition with a stopwatch (0.71, 95% CI 0.283–0.882) is lower than those reported in previous studies that investigated test–retest reliability in stroke [26] (0.985, 95% CI 0.973–0.992), community-dwelling older adults [27] (0.940, 95% CI 0.90–0.96), and primary total knee arthroplasty [46] (0.942).

Our findings might be due to the symptomatology of FM [47], characterized by widespread pain and fatigue, which can fluctuate in intensity and severity over time [48,49]. For this reason, a person’s symptoms and level of functioning can vary daily and affect the physical fitness performance. In this regard, previous studies highlighted what can affect performance in physical fitness tests [50,51]. The same rationale can also affect cognitive function [52], including attention, memory, and information processing. These factors may have contributed to the variability of results between the test and retest. Nevertheless, the ICC values obtained in the present study are acceptable, so the test analyzed seems to be stable enough to be used in the characterization of people with FM.

The optimal time interval between tests may vary depending on the construct being measured, the stability of the construct over time, and the target population [53]. For this study, a seven-day period was selected, the same as previous studies conducted in people with FM [11,18,38,39], to minimize the impact of potential confounding variables, such as recovery or learning effects [54]. However, other studies have used shorter test–retest times to assess test–retest reliability on the 3MBWT [26,27,28,29]. In addition, the tests (TUG and 3MBWT), as well as the conditions (single and dual-task), were randomized to ensure that the order of administration may not bias the results. In this way, obtaining more accurate and reliable results is possible by eliminating biases and reducing the influence of external factors that may affect the results.

As expected, the ICC values were slightly higher when using Chronopic versus stopwatch, since the use of a manual stopwatch adds human variability to the measurement by the evaluator [9,55]. Therefore, using an automatic timer can be a cost-effective alternative to assess performance in the 3MBWT in both single and dual conditions. However, although the ICC values obtained by the stopwatch are slightly lower than those obtained by the Chronopic, our data suggest that the use of a manual stopwatch could be also very useful for this test, since it yields relatively good reliability values in both single (0.71, 95% CI 0.283–0.882) and dual-task conditions (0.89, 95% CI 0.718–0.954) (see Table 2). Similarly, the lower ICC obtained in the single and dual-task conditions in the test performance was probably due to human error experienced when using a manual stopwatch. This may be suggested since the scores between the stopwatch and the Chronopic differed slightly, unlike the values obtained in the new test. Nevertheless, the ICC values obtained are classified as good to excellent (see Table 3).

Our study also provided the SEM and MDC values of the 3MBWT under all conditions and devices. These values are important for interpreting the results of the 3MBWT, because they can help clinicians and researchers to determine if there are meaningful changes in the performance of this test. Furthermore, the estimate of random variation in the data (SEM) and the minimum detectable change (MDC) are both lower when the Chronopic is used. Previous research has also observed this trend assessing test–retest reliability under the single condition in TUG and 30 s chair stand test [9,55]. Bland–Altman plots were also reported for a more comprehensive analysis of the results, showing bias and limits of agreement.

In our study, the TUG test was used to test the validity of the 3MBWT since it is a tool frequently used in clinical practice [56,57], showing high reliability in FM in both single [9] and dual-task conditions [11]. Although the TUG test does not contemplate walking backward, as previous studies did [26,27,28,29], in the present study we have used the TUG test to conduct the concurrent validity. Moreover, this test has obtained the highest relationship values in most cases [26,27,29]. Significant correlations between 3MBWT and TUG tests were obtained, which can be considered relevant since the TUG test comprises movements that can occur in activities of daily living, including walking, turning, and sitting [37]. In addition, correlation analyses showed a strong correlation in the single condition between the 3MBWT and TUG test measured with a stopwatch, as in previous studies in stroke [26], community-dwelling older adults [27], and adults [23]. In our study, the correlation in single conditions between 3MBWT and TUG measured through the automatic timer also showed strong correlation values. Similarly, the correlation in the dual-task conditions also obtained strong values in the test and retest measured by a stopwatch and a Chronopic.

Our results also found a positive correlation between the 3MBWT performance and the impact of FM obtained through the FIQR questionnaire. Strong to moderate correlations were found between FIQR total score and the performance obtained in single and dual-task conditions using manual stopwatch and Chronopic. Additionally, correlations between FIQR dimensions and 3MBWT have been reported. In this line, the strongest correlations were found in the symptoms dimension, followed by overall impact, where moderate correlations were also found. However, function dimension did not show significant correlation with 3MBWT performance in most cases. These findings could indicate that performance on the 3MBWT does not correlate well with the actions included in the function dimension by including activities that are not highly associated with walking backward (i.e., combing hair, preparing food, shopping) but do correlate with symptomatology and overall impact. Nevertheless, future studies should corroborate this hypothesis.

Previous studies have highlighted the importance of assessing backward gait in older adults [24,25], showing a reduction in performance in different parameters when comparing to young and middle-aged adults. In this regard, during backward gait, greater neuromuscular and proprioceptive control is required, in addition to faster and more frequent balance corrections due to the elimination of visual feedback [22,58]. Additionally, it has been shown that walking backward shows greater or equal sensibility than walking forward, and it is strongly related to the risk of falling [23]. In this sense, assessing backward gait, via the 3MBWT, in FM may be of interest to clinicians to observe changes in mobility and balance due to the balance impairment manifested in this population [59]. Thus, a previous study [60] conducted a physical exercise intervention based on walking backward in community-dwelling older adults. Given these reasons and the strong relationships between the impact of the disease and the performance obtained in the 3MBWT, this test can be used as a useful tool in FM populations to assess exercise-based interventions.

The present study has some limitations. In this regard, the relatively small sample size did not allow us to generalize results to all women with FM. Moreover, only women were included in this cross-sectional study. Thus, results cannot be extrapolated to men with this disease. Therefore, it would be interesting to extend the sample with different age ranges, which allow us to establish cut-off points.

## 5. Conclusions

The results obtained from this study show that the 3MBWT is a reliable tool under the single and dual-task conditions in women with FM. It shows higher reliability values when time is taken using a Chronopic. This test also shows high concurrent validity with the TUG test, and its performance is related to the impact of the disease. These results may help clinicians and researchers in the assessment of balance and functional mobility and to interpret the effect of interventions in this population.

## Figures and Tables

**Table 1:** Descriptive characteristics of the participants.

Variables (N = 21)	Mean (SD)
Age (years)	52.48 (5.99)
Height (cm)	160.10 (0.07)
Weight (kg)	73.50 (14.37)
BMI (kg/m^2^)	28.67 (5.53)
FIQR	59.68 (22.07)
FIQR-Function	16.06 (7.74)
FIQR-Overall impact	10.71 (6.90)
FIQR-Symptoms	32.90 (9.77)
Falls in the last six months (number)	0.95 (1.20)

Abbreviations: N, sample; SD, standard deviation; BMI, body mass index, FIQR, Fibromyalgia Impact Questionnaire Revised.

**Table 2:** Reliability of the 3MBWT under single and dual-task conditions.

Variables		Test	Retest	ICC (95% CI)	SEM	%SEM	MDC	%MDC
3MBWT (s)	Stopwatch	2.90 (0.87)	2.85 (1.13)	0.71 (0.283–0.882)	0.54	18.73	1.49	51.92
	Chronopic	2.80 (0.88)	2.75 (1.09)	0.85 (0.619–0.937)	0.38	13.75	1.06	38.11
3MBWT DT (s)	Stopwatch	3.21 (0.95)	2.81 (1.10)	0.89 (0.718–0.954)	0.34	11.29	0.94	31.31
	Chronopic	3.06 (1.03)	2.90 (1.03)	0.93 (0.817–0.970)	0.27	9.14	0.76	25.35

Abbreviations: 3MBWT, 3 m backward test; s, seconds; SD, standard deviation; ICC, intraclass correlation coefficient; CI, confidence interval; SEM, standard error of measurement; MDC, minimal detectable change; DT, dual-task.

**Table 3:** Reliability of the 3MBWT under single and dual-task conditions using stopwatch and Chronopic in the test and retest.

Variables		Stopwatch Mean (SD)	Chronopic Mean (SD)	ICC (95% CI)
3MBWT (s)	Test	2.90 (0.87)	2.80 (0.88)	0.92 (0.811–0.969)
	Retest	2.85 (1.13)	2.75 (1.09)	0.974 (0.937–0.990)
3MBWT DT (s)	Test	3.21 (0.95)	3.06 (1.03)	0.91 (0.779–0.964)
	Retest	2.81 (1.10)	2.90 (1.03)	0.974 (0.937–0.990)

Abbreviations: 3MBWT, 3 m backward test; s, seconds; SD, standard deviation; ICC, intraclass correlation coefficient; CI, confidence interval; DT, dual-task.

**Table 4:** Concurrent validity between 3MBWT and TUG under single and dual-task conditions, in test and retests, with stopwatch and Chronopic.

**Stopwatch**			
		Test Condition	Retest Condition
	Variables	TUG	TUG
**Single**	3MBWT	0.735 ***	0.831 ***
**Dual**	3MBWT	0.679 ***	0.875 ***
**Chronopic**			
		Test Condition	Retest Condition
	Variables	TUG	TUG
**Single**	3MBWT	0.834 ***	0.794 ***
**Dual**	3MBWT	0.845 ***	0.906 ***

Abbreviations: 3MBWT, 3-m backward test; TUG, timed up and go. *** *p*-value < 0.001.

**Table 5:** Correlation between 3MBWT and FIQR under single and dual-task conditions, in test and retest, with stopwatch and Chronopic.

**Stopwatch**					
	Variables	FIQR	FIQR-Function	FIQR-Overall impact	FIQR-Symptoms
**Single**	3MBWT (test)	0.488 *	0.273	0.488 *	0.543 *
	3MBWT (retest)	0.659 ***	0.461 *	0.652 **	0.663 ***
**Dual**	3MBWT (test)	0.527 *	0.396	0.513 *	0.514 *
	3MBWT (retest)	0.614 ***	0.389	0.630 **	0.634 **
**Chronopic**					
	Variables	FIQR	FIQR-Function	FIQR-Overall impact	FIQR-Symptoms
**Single**	3MBWT (test)	0.654 ***	0.448 *	0.558 **	0.729 ***
	3MBWT (retest)	0.577 ***	0.325	0.624 **	0.604 **
**Dual**	3MBWT (test)	0.532 *	0.356	0.444 *	0.607 **
	3MBWT (retest)	0.636 ***	0.394	0.631 **	0.679 ***

Abbreviations: 3MBWT, 3-m backward test; FIQR, fibromyalgia impact questionnaire revised. * *p*-value < 0.05; ** *p*-value < 0.01; *** *p*-value < 0.001.

## Data Availability

Data are available under reasonable request to the corresponding author.
